# Step-Initiation Deficits in Children with Faulty Posture Diagnosed with Neurodevelopmental Disorders during Infancy

**DOI:** 10.3389/fped.2017.00239

**Published:** 2017-11-03

**Authors:** Magdalena Stania, Alina Sarat-Spek, Teresa Blacha, Beata Kazek, Kajetan J. Słomka, Ewa Emich-Widera, Grzegorz Juras

**Affiliations:** ^1^Faculty of Physiotherapy, Department of Physiotherapy Basics, Jerzy Kukuczka Academy of Physical Education, Katowice, Poland; ^2^The Independent Public Clinical Hospital No. 6 of the Medical University of Silesia in Katowice, The John Paul II Upper Silesia Child Health Centre, Katowice, Poland; ^3^Persevere – Child Development Support Center, Katowice, Poland; ^4^Department of Human Motor Behavior, Jerzy Kukuczka Academy of Physical Education, Katowice, Poland; ^5^Department of Pediatric Neurology, Medical University of Silesia, Katowice, Poland

**Keywords:** step initiation, children, neurodevelopmental disorders, postural balance, faulty posture

## Abstract

**Background:**

Early detection of movement deficits during step initiation will facilitate the selection of the optimal physiotherapy management strategy. The main aim of the study was to assess potential differences in step initiation between 5- and 6-year-old children with faulty posture who had been diagnosed with neurodevelopmental disorders during infancy and healthy children.

**Methods:**

The experimental group consisted of 19 children aged 5–6 years with faulty posture, who had been diagnosed with neurodevelopmental disorders during infancy and were given physiotherapy in the first year of their lives. The control group comprised 19 nursery school children aged 5–6 years with no postural defects, no history of postural control or movement deficits, and no physiotherapy interventions in the first year of their lives. Step initiation was performed on force platforms under various conditions, i.e., with and without an obstacle, stepping up onto a platform placed at a higher level, stepping down onto a platform placed on a lower level. The recording of center of foot pressure (COP) displacements was divided into three phases: phase 1 (P1)—quiet standing before step initiation, phase 2 (P2)—transit, phase 3 (P3)—quiet standing until measurement completion.

**Results:**

The Tukey *post hoc* test showed that the means of sway range (raCOP) and mean velocity (vCOP) in sagittal (_AP_) plane for phase 1 and vCOP in frontal (_ML_) plane for phase 3 registered in the step-up trial were significantly higher (*p* < 0.05) in children with faulty posture compared to children with typical development. P1vCOP_ML_, P3vCOP_AP_, P3raCOP_ML_, and P3vCOP_ML_of the step-down trial were also significantly higher in children with faulty posture (*p* < 0.05).

**Conclusion:**

Inclusion of functional movement exercises (stair-walking tasks) in physiotherapy interventions for children with postural defects seems well justified.

The trial was registered in the Australian and New Zealand Clinical Trials Registry (no. ACTRN12617001068358).

## Introduction

Postural control during step initiation is a complex motor task involving forward weight-shift associated with transfer from static to dynamic balance (1). Normal gait initiation depends on the activity of skeletal muscles, ground reaction forces, center-of-pressure (COP), and centre of mass displacements, involving joint motions (2). Prior to a voluntary movement, any potential imbalance is counteracted by anticipatory postural adjustments based on feed-forward activation of postural muscles (3).

The ability of a child to maintain or regain balance during a variety of motor activities is of fundamental importance for efficient motor behavior. Maintenance of balance is inseparably associated with motor learning, which, in turn, is based on open-loop and closed-loop postural control mechanisms (4). Although several reports on static postural stability of children have recently been published (5–7), further research is needed to study postural control in children during everyday activities including walking up and down stairs or stepping over an obstacle (e.g., threshold). Objective measurement tools, e.g., posturography platforms, facilitate diagnostic reliability; however, new analysis approaches of the COP trajectory in postural sway continue to be explored (8). A reliable diagnosis of postural control in children can lead to identification of the individuals at risk for falling. Falls are the leading cause of emergency department admissions in nursery school children, 12% of which are falls from stairs (9).

The ability to initiate a step is frequently disregarded in physiotherapy whereas all movement deficits should be identified early enough to prevent the negative effects thereof on the child’s motor development. Early detection of perturbed step initiation would facilitate the selection of the optimal physiotherapy management strategy. The available literature reports analyze gait initiation in children with unilateral idiopathic clubfoot (10), cerebral palsy (11), and Rett syndrome (12). The manifestations of gait disturbances include shorter step length, faster gait speed, and increased period of double-limb support (10, 12). Step initiation in children with postural defects who had been diagnosed with neurodevelopmental disorders during infancy has gained increasing interest. Poor posture is a common finding among young people—a study among child and adolescent population revealed at least one body posture defect in 67.9% of the participants (13).

According to the neurodevelopmental approach, central nervous system dysfunctions result in abnormal postural tone and antigravity mechanism impairment—hence the development of compensatory movement patterns. Inadequate sensorimotor stimuli processing and input to relevant cortical areas lead to dysfunction of the postural control system and locomotor system (14). Therefore, children with faulty posture, diagnosed with neurodevelopmental disorders during infancy are expected to present deficits in step initiation.

The main aim of the study was to assess potential differences in step initiation between 5 and 6 year old children with faulty posture who had been diagnosed with neurodevelopmental disorders during infancy and healthy children. It was hypothesized that faulty posture is related to compromised step-related movement deficits. It was also speculated that posturography parameters characterizing step initiation in 5- to 6-year-old children depend on difficulty of the step-initiation task.

## Materials and Methods

A case–control study was designed to compare step initiation in children with normal and faulty postures. The experiment was carried out in July 2017 in the Pediatric Hospital No. 6, Medical University of Silesia and two nursery schools in Katowice, Poland. The trial was registered in the Australian and New Zealand Clinical Trials Registry (no. ACTRN12617001068358).

### Subjects

The cases were recruited from children inhabiting the Silesian region in Poland.

The experimental group consisted of 19 children (7 boys and 12 girls) aged 5–6 years (the mean age was 5.4 ± 0.3) with faulty posture identified on neurological and physiotherapy examinations, who had been diagnosed with neurodevelopmental disorders during infancy and were given physiotherapy in the first year of their lives. The following postural deformities were included in the experimental group: shoulder and scapular asymmetry, abnormal curvatures of the spine, incorrect knee alignment, foot arch deformity, foot deformity, and abnormalities in the antigravity mechanism (low muscle tone, muscle tone asymmetry). The control group comprised 19 nursery school children (8 boys and 11 girls) aged 5–6 years (the mean age was 5.6 ± 0.5) with no postural defects on neurological and physiotherapy examinations, no history of postural control or movement deficits and no physiotherapy interventions in the first year of their lives.

No significant differences in physical characteristics were found between the experimental and control groups at baseline. The mean body mass was 20.1 ± 4.9 kg and the mean height was 116.2 ± 9.1 cm.

The exclusion criteria were genetic disorders, progressive encephalopathy, congenital abnormalities of the central nervous system, infantile cerebral palsy, no consent of the child, and/or parents (guardians) to participate in the study.

An informed written consent was given by all parents. The study was approved by an ethics committee of the Institutional Review Board of Medical University of Silesia, Katowice, Poland (KNW/0022/KB1/54/16).

### Step Initiation Assessment

The measurement station consisted of two (A and B) force AMTI platforms (AccuGait), charge amplifier, and computer. Digital output from the platform was recorded using AMTI’s NetForce software with recording frequency of 100 Hz. The off-line raw data were low-pass filtered at 6 Hz using dual-pass Butterworth digital filter with MATLAB software (Mathworks, Natic, MA, USA) (15).

Quiet standing was the starting position in all trials with feet comfortably aligned, arms along the trunk and eyes looking straight ahead.

Assessment of step initiation comprised four trials:

1. Trial 1 (unperturbed transit)—three repetitions: quiet standing on platform A for 15 s, then changing to Platform B (one step) followed by quiet standing until measurement completion. The distance between platforms: 10 cm.
2. Trial 2 (perturbed transit)—three repetitions: quiet standing on Platform A for 15 s, then changing to Platform B (one step) followed by quiet standing until measurement completion. A 15-cm high and 4 cm thick obstacle was inserted between platforms which yielded higher foot clearance and higher cognitive demand for the participants.
3. Trial 3 (step-up)—three repetitions: quiet standing on Platform A for 15 s, then changing to Platform B (one step up) followed by quiet standing until measurement completion. Platform B was placed on a 17-cm base directly at the edge of Platform A.
4. Trial 4 (step-down)—three repetitions: quiet standing on Platform B for 15 s, then changing to Platform A (one step down) followed by quiet standing until measurement completion. Platform B was placed on a 17-cm base directly at the edge of Platform A.

Platform changing started each time at a sound cue. Children participated in all trials barefoot and at their own tempo. They were not instructed which foot to start with. However, each trial was preceded by a rehearsal so that the participants knew how to perform.

The recording of center of foot pressure displacements was divided into three phases: phase 1—quiet standing before step initiation, phase 2—transit, and phase 3—quiet standing until measurement completion. The recording was divided into phases using an algorithm whose main elements was foot contact with the platform and the limit of momentary COP displacement; beyond that point exit from stability or stability gain was observed. Stability is defined as body sway where momentary COP displacement does not exceed mean COP displacement plus three SDs. For phase 1, mean COP and SD was calculated based on measurements made within the first 5 s of the test; For phase 3—based on the last 5 s of the test. The following variables of COP displacement were calculated for phases 1 and 3: sway range (raCOP) (centimeters) and mean velocity of COP (vCOP) (centimeters per second) for sagittal (_AP_) and frontal (_ML_) plane. The following variables were determined for phase 2:

D1—time from exit from stability state until foot rest on the other platform (seconds),
D2—time from raising the foot from the first platform until gaining stability on the other platform (seconds),
Transit time (phase 2)—time from exit from stability state until gaining post-transit stability; Transit time = the sum of D1 + double-support period + D2 (seconds). Double-support period is when each foot is in contact withone of the platforms (seconds).

### Statistical Analysis

Considering the probability of a type I error alpha = 0.05, target power of 1-beta = 0.80 and a 25% minimum significant difference between the means of the study parameters, the resultant minimum sample size in each group was 18 participants. Two additional participants were recruited to make up for potential dropouts. The study participants were assigned to groups of 19 children each.

Normality of distribution was checked using the Shapiro–Wilk test; the assumption of homogeneity of variance was tested with Levene’s test. The method of statistical analysis was two-way ANOVA with a 2 × 4 factorial design (group × testing condition). *Post hoc* comparisons were performed using the Tukey test. Repeated measures one-way ANOVA was used to compare each group’s means under particular testing conditions, with a Bonferroni correction as the *post hoc* test. *p* ≤ 0.05 was defined as the level of statistical significance in all tests.

## Results

The two-way interactions group × testing conditions were not significant for any of the study variables during phase 1, 2, and 3 (*p* > 0.05).

**Figure 1:**
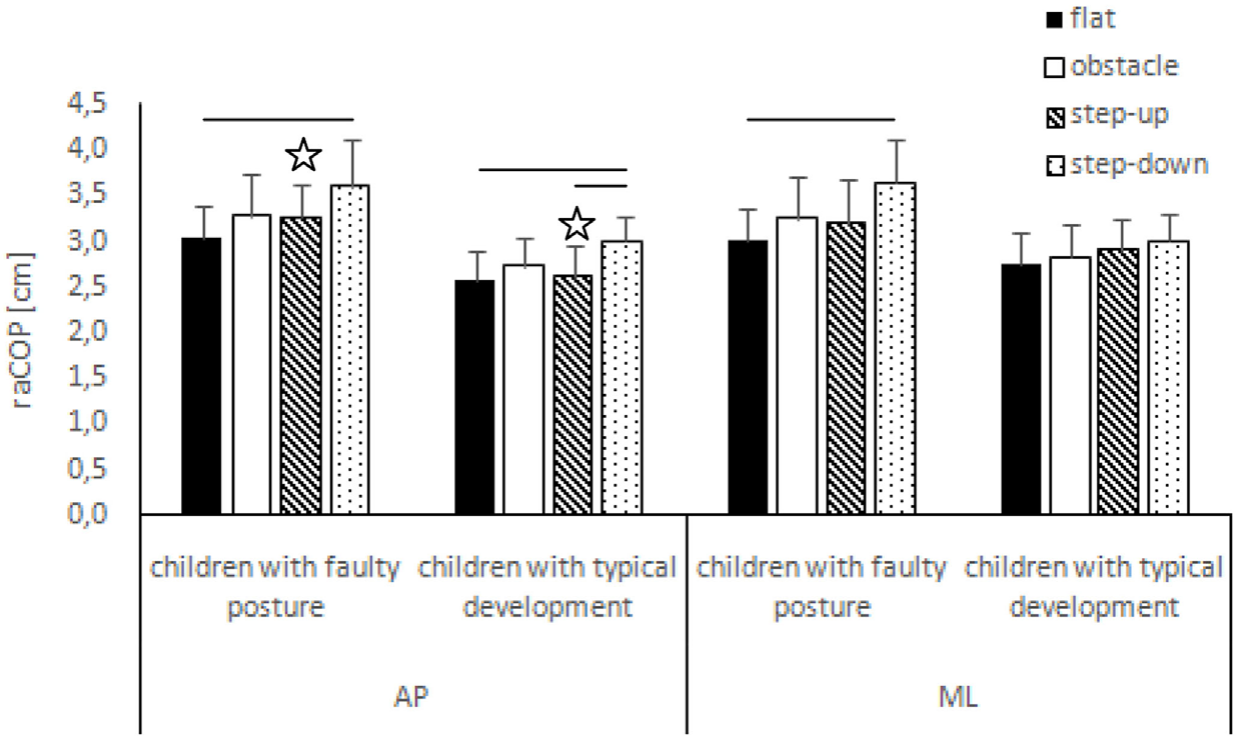
Study and control groups—mean raCOP changes (±SDs marked as error bars) in the sagittal (AP) and frontal (ML) planes during quiet standing before step initiation depending on testing conditions (phase 1). Stars indicate significant intergroup differences [two-way ANOVA with a 2 × 4 factorial design (group × testing condition)] with Tukey *post hoc* test; horizontal bars indicate statistically significant differences within the groups (repeated measures one-way ANOVA with a Bonferroni correction as the *post hoc* test).

**Figure 2:**
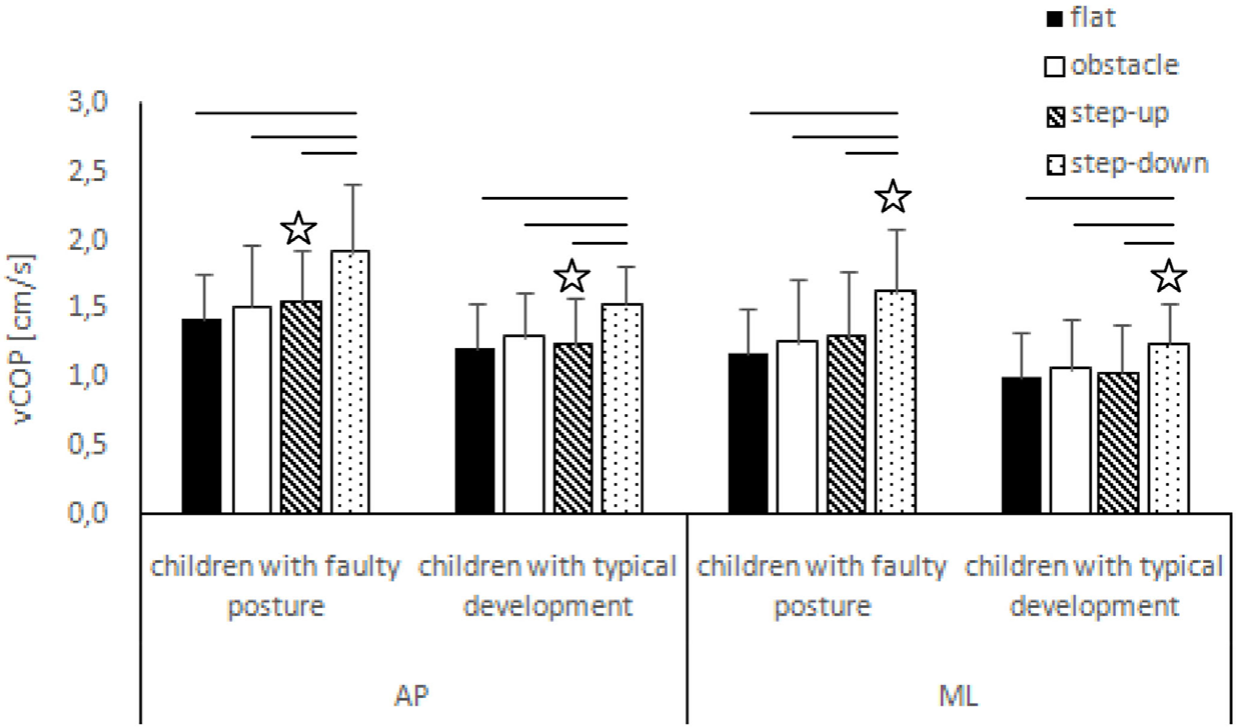
Study and control groups—mean vCOP changes (±SDs marked as error bars) in the sagittal (AP) and frontal (ML) planes during quiet standing before step initiation depending on testing conditions (phase 1). Stars indicate significant intergroup differences [two-way ANOVA with a 2 × 4 factorial design (group × testing condition)] with Tukey *post hoc* test; horizontal bars indicate statistically significant differences within the groups (repeated measures one-way ANOVA with a Bonferroni correction as the *post hoc* test).

**Figure 3:**
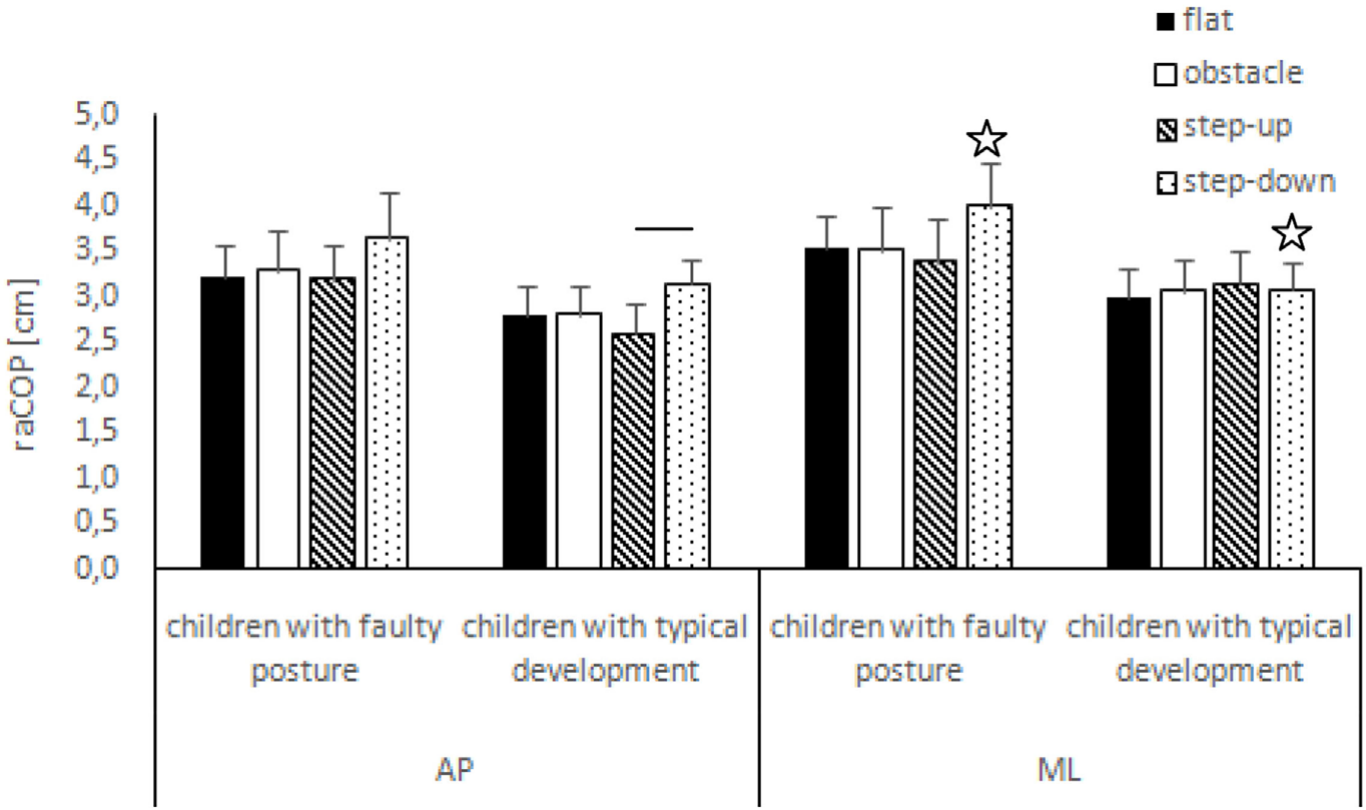
Study and control groups—mean raCOP changes (±SDs marked as error bars) in the sagittal (AP) and frontal (ML) planes during quiet standing after step initiation depending on testing conditions (phase 3). Stars indicate significant intergroup differences [two-way ANOVA with a 2 × 4 factorial design (group × testing condition)] with Tukey *post hoc* test; horizontal bars indicate statistically significant differences within the groups (repeated measures one-way ANOVA with a Bonferroni correction as the *post hoc* test).

**Figure 4:**
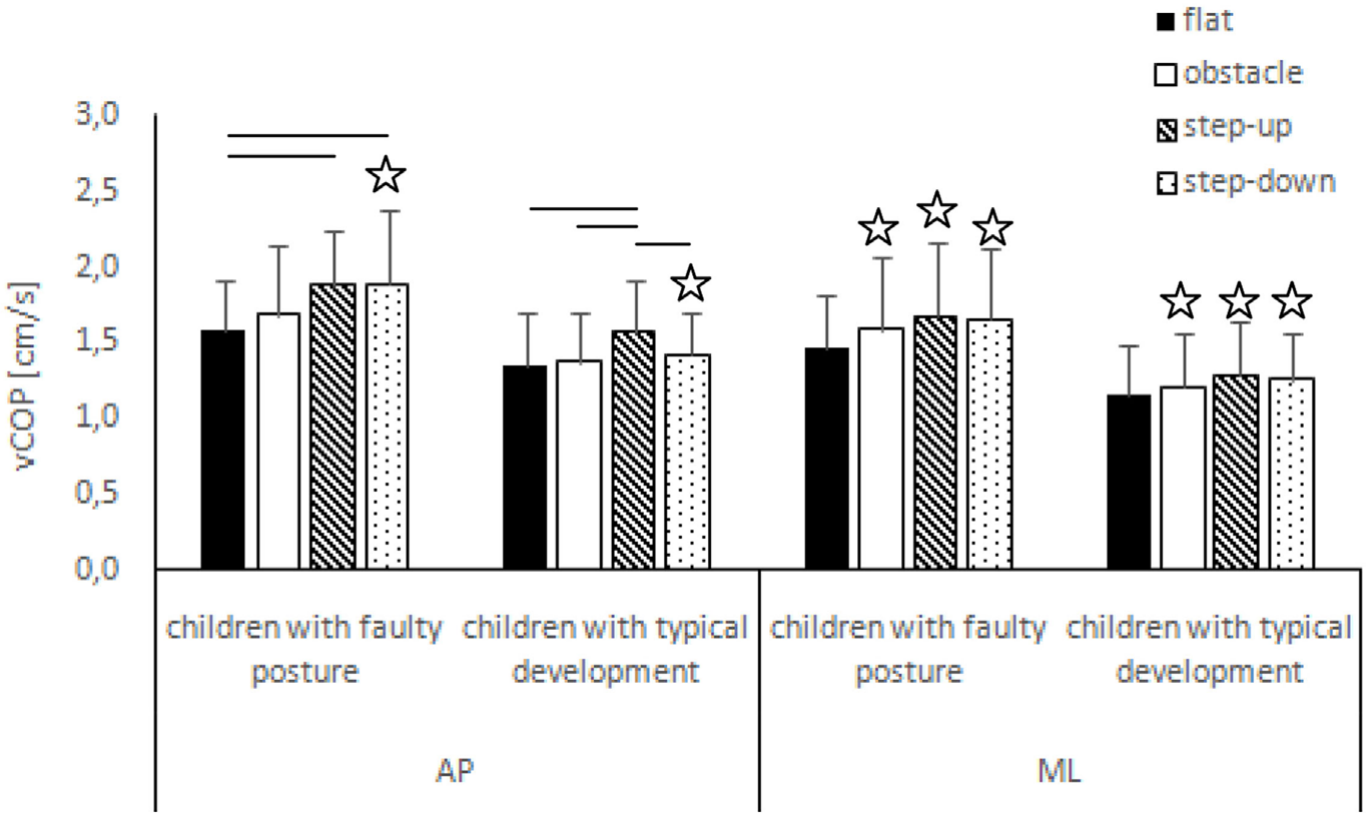
Study and control groups—mean vCOP changes (±SDs marked as error bars) in the sagittal (AP) and frontal (ML) planes during quiet standing after step initiation depending on testing conditions (phase 3). Stars indicate significant intergroup differences [two-way ANOVA with a 2 × 4 factorial design (group × testing condition)] with Tukey *post hoc* test; horizontal bars indicate statistically significant differences within the groups (repeated measures one-way ANOVA with a Bonferroni correction as the *post hoc* test).

The two-way ANOVA revealed a group effect on phase 1 COP sway range and velocity for sagittal plane, phase 1 COP velocity for frontal plane, phase 2 COP velocity for sagittal plane, and phase 2 COP sway range and velocity for frontal plane. The Tukey *post hoc* test showed that the means of phase 1 COP sway range (*p* = 0.04) and velocity (*p* = 0.02) for sagittal plane and phase 3 COP velocity for frontal plane (*p* = 0.04) obtained for the step-up trial were significantly higher in children with faulty posture compared to children with typical development. Phase 1 COP velocity for frontal plane (*p* = 0.003), phase 3 COP velocity for sagittal plane (*p* = 0.003), and phase 3 COP sway range (*p* = 0.02) and velocity (*p* = 0.04) for frontal plane of the step-down trial were also significantly higher in children with faulty posture. The *post hoc* analysis showed that the phase 3 COP velocity for frontal plane, obtained during obstacle crossing, was significantly higher in children with faulty posture (*p* = 0.04) (Figures 1–4).

### Impact of Testing Conditions on Trial Performance—Intragroup Comparison

#### Phase 1

##### Children with Faulty Posture

In children with faulty posture, one-way ANOVA revealed a significant impact of testing conditions on COP sway range and velocity for both sagittal and frontal planes (*p* < 0.05). A Bonferroni correction confirmed significantly higher COP sway range for sagittal and frontal planes during quiet standing in the step-down trial compared to unperturbed transit; *p* = 0.006 and *p* = 0.007, respectively. Step-down COP velocity for both sagittal and frontal planes was significantly higher compared to all other trials (*p* < 0.05).

##### Children with Typical Development

In children with typical development, one-way ANOVA revealed a significant impact of testing conditions on COP sway range and velocity for sagittal plane and COP velocity for frontal plane (*p* < 0.05). *Post hoc* analysis confirmed significantly higher COP sway range in sagittal plane in the step-down trial compared to unperturbed transit and step-up trials; *p* = 0.005 and *p* = 0.01, respectively. COP velocity for both sagittal and frontal planes were significantly higher during post-step-down quiet standing compared to all other trials (*p* < 0.05) (Figures 1 and 2).

#### Phase 2

##### Children with Faulty Posture

In children with faulty posture, one-way ANOVA revealed a significant impact of testing conditions on transit time, which was significantly longer in perturbed compared to unperturbed transit (*p* = 0.02). D2 (time from raising the foot from the first platform until gaining stability on the other platform) of the step-up trial was significantly longer compared to unperturbed transit (*p* = 0.03).

##### Children with Typical Development

**Figure 5:**
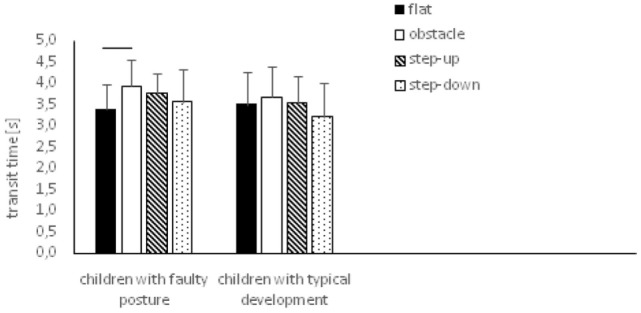
Study and control groups—mean transit time changes (±SDs marked as error bars) during step initiation depending on testing conditions (phase 2). Horizontal bars indicate statistically significant differences within the groups (repeated measures one-way ANOVA with a Bonferroni correction as the *post hoc* test).

In children with typical development, one-way ANOVA did not reveal any significant impact of testing conditions on transit time (*p* = 0.054), D1 (time from exit from stability state until foot rest on the other platform) (*p* = 0.154) and D2 (*p* = 0.46) (Figure 5).

#### Phase 3

##### Children with Faulty Posture

In children with faulty posture, one-way ANOVA revealed a significant impact of testing conditions on COP sway range and velocity for sagittal plane (*p* < 0.05). Bonferroni correction confirmed significantly higher COP velocity for sagittal plane in the step-up and step-down trials compared to unperturbed transit; *p* = 0.006 and *p* = 0.005, respectively.

##### Children with Typical Development

In children with typical development, one-way ANOVA revealed a significant impact of testing conditions on COP sway range and velocity for sagittal plane (*p* < 0.05). Step-down COP sway range for sagittal plane was significantly higher compared to step-up trial (*p* = 0.006). COP velocity for sagittal plane was significantly higher in the step-up compared to all other trials (*p* < 0.05) (Figures 3 and 4).

## Discussion

Among the key issues of neurodevelopmental therapy is not only whether a child is capable of performing a motor task; the quality of performance is also important. The main aim of the study was posturographic assessment of potential differences in step initiation between 5 and 6 year old children with faulty posture who had been diagnosed with neurodevelopmental disorders during infancy and healthy children. Children with faulty posture and healthy participants exhibited the greatest range and velocity of COP displacement in quiet standing in the step-down trial, indicating considerable difficulty of this motor task for 5- to 6-year olds. Protopapadaki et al. (16) had a contrary observation in a group of healthy subjects aged 18–39 years, for whom stair ascent was a more demanding biomechanical task compared to stair descent. This discrepancy probably stems from morphological, biomechanical, and ontogenetic determinants. In general, stair-walking tasks require greater ground reaction forces and knee force moments in comparison to level walking (17). As expected, unperturbed transit between platforms was the simplest task for both groups. No intergroup differences were revealed with respect to step initiation on a flat surface suggesting that postural defects do not hamper motor performance during simple motor tasks. In children with faulty posture, compensatory mechanisms might mitigate or overcome deficits (10). Slobounov and Newell (18) observed compensatory movement pattern strategies for the recovery of postural stability in a group of 5-year olds and our study participants were 5–6 years old.

According to Boonyong et al. (19), stepping over an obstacle is a more challenging task than unperturbed level walking as evidenced by reduced gait velocity with longer stride time and stride length and a wider step width. Our study also revealed longer transit time in perturbed transit (Trial 2) compared to the remaining trials; however, no significant intergroup differences were noted. Deconinck et al. (20) observed that a majority of spatiotemporal gait parameters of obstacle crossing did not differ significantly between typically developing children and those with Developmental Coordination Disorder. The authors concluded both groups exhibited satisfactory anticipatory control, adequate visual guidance, and the same obstacle crossing technique, i.e., lengthening the distance of the lead step and shortening the trail step (20).

Our analysis showed a significantly greater range and velocity of COP displacement in quiet standing prior to/after the step-up and step-down trials in children with faulty posture compared to healthy participants. Hence, it seems that postural defects are associated with less efficient postural control during more difficult motor tasks. According to the neurodevelopmental approach, postural defects result from abnormal postural tone which, in turn, causes postural stability impairment. The locomotor system is inefficient, and hence, the child uses acquired compensatory movement patterns (14).

Postural control plays a fundamental role in motor behavior including gait initiation. Depending on age and phase of ontogenetic development, children apply different balance control strategies (21). Young children initially adopt a variety of balance control strategies and select the optimal one at a later stage. A balance strategy involves the choice of a stable reference frame around which movements are built up and a gradual mastery of the degrees of freedom of body joints. The key reference frames are the pelvis to allow better control of the center of gravity or the head to achieve better visual and vestibular processing (22). Reduction of “noise” in the postural control system and increased ability to use multisensory inputs contribute to continued postural control development, but these processes change in a non-homogeneous manner (6). Demura et al. (5) concluded that the changes of postural sway characteristics in young children with growth might largely be influenced by nervous system maturation rather than by physique.

Physiotherapy interventions in children with postural defects should focus on improvement of sensorimotor control, increasing the efficiency of musculofascial bands, correction of body asymmetry, and improvement of core stability. Restoration of normal movement patterns and improvement in the function of the antigravity mechanism help correct body posture (23). Based on our results, we recommend adding functional movement exercises including stair-walking tasks.

## Ethics Statement

The study was approved by an ethics committee of the Institutional Review Board of Medical University of Silesia, Katowice, Poland (Approval date: 24/05/2016, ethics approval number: KNW/0022/KB1/54/16).

## Author Contributions

Conceived and designed the study: AS-S, TB, KJS, EE-W, and GJ; participated in data collection: MS, AS-S, TB, BK, and EE-W; statistical analysis: KJS; data analysis: MS, KJS, and GJ; wrote the paper: MS and GJ; manuscript editing: MS, AS-S, TB, BK, KJS, EE-W, and GJ. All authors approved the final version of manuscript.

## Conflict of Interest Statement

The authors declare that the research was conducted in the absence of any commercial or financial relationships that could be construed as a potential conflict of interest.

## Abbreviations

AP, sagittal plane; COG, center of gravity; COM, centre of mass; COP, center of pressure; ML, frontal plane; P1, phase 1; P2, phase 2; P3, phase 3; raCOP, sway range of COP displacements; vCOP, velocity of COP displacements.
